# Alterations of the NK cell pool in HIV/HCV co-infection

**DOI:** 10.1371/journal.pone.0174465

**Published:** 2017-04-05

**Authors:** Dominik J. Kaczmarek, Pavlos Kokordelis, Benjamin Krämer, Andreas Glässner, Franziska Wolter, Felix Goeser, Philipp Lutz, Carolynne Schwarze-Zander, Christoph Boesecke, Christian P. Strassburg, Jürgen K. Rockstroh, Ulrich Spengler, Jacob Nattermann

**Affiliations:** 1 Department of Internal Medicine I, University of Bonn, Bonn, Germany; 2 German Center for Infection Research (DZIF), Bonn, Germany; Karolinska Institutet Department of Medicine Solna, SWEDEN

## Abstract

**Background:**

A relevant proportion of human immunodeficiency virus (HIV) infected patients is co-infected with the hepatitis C virus (HCV). HCV co-infection in HIV-positive patients is associated with faster progression of liver disease in comparison to HCV mono-infection. Natural killer (NK) cells critically modulate the natural course of HCV infection. Both HIV and HCV mono-infection are associated with alterations of the NK cell pool. However, little data is available concerning phenotype and function of NK cells in HIV/HCV co-infection.

**Methods:**

A total of 34 HIV/HCV co-infected, 35 HIV and 39 HCV mono-infected patients and 43 healthy control persons were enrolled into this study. All HIV-positive patients were under effective antiretroviral therapy. NK cell phenotype, IFN-γ production and degranulation were studied by flow cytometry.

**Results:**

NK cell frequency in HIV/HCV co-infection was significantly lower than in healthy individuals but did not differ from HIV and HCV mono-infection. HIV/HCV co-infection was associated with significantly decreased expression of the maturation/differentiation markers CD27/62L/127 on NK cells but increased expression of CD57 compared to healthy controls. Of note, expression also differed significantly from HCV mono-infection but was similar to HIV mono-infection, suggesting a pronounced impact of HIV on these alterations. Similar findings were made with regard to the NK cell receptors NKG2A/C and NKp30. More importantly, NK cells in co-infection displayed a highly impaired functional activity with significantly lower IFN-γ production and degranulation than in healthy donors as well as HIV and HCV mono-infection, suggesting a synergistic effect of both viruses.

**Conclusions:**

Our data indicate that HIV/HCV co-infection is associated with significant alterations of the NK cell pool, which might be involved in the rapid progression of liver disease in co-infected patients and which mainly reflect alterations observed in HIV mono-infection.

## Introduction

Due to similar transmission routes of infection a relevant proportion of human immunodeficiency virus (HIV)-positive patients is co-infected with the hepatitis C virus (HCV)[1]. HIV/HCV co-infection is associated with a faster progression to liver fibrosis and cirrhosis, resulting in higher mortality compared to HCV mono-infected individuals[2–8]. Accordingly, liver-associated mortality has become a major cause of death in HIV-positive (HIV(+)) persons under combined anti-retroviral therapy (cART)[3].

Incomplete restoration of the immune system in HIV patients despite effectively blocked HIV replication is considered to importantly contribute to this phenomenon[9–12]. In this context, persistent dysregulation of the natural killer (NK) cell pool is of especial interest, as NK cells have been shown to effectively block HCV replication[13,14] and to display an anti-fibrotic activity[15].

Both HIV and HCV mono-infection are associated with significant perturbations of NK cells. For instance, a reduction of absolute NK cell numbers was found in HIV(+) as well as in HCV(+) patients[16,17] and both HIV and HCV mono-infection as well as HIV/HCV co-infection have been observed to be associated with the appearance of a highly dysfunctional subset of CD56^-^ CD16^+^ NK cells, characterized by a poor cytotoxic activity[18–23]. In addition, both viral infections are characterized by altered expression patterns of activating NK cell receptors (NKR)[11,24,25] although reports on NKR expression in chronic hepatitis C are controversial[24–31]. Besides these shared alterations in NK cell phenotype and functions observed in HIV and HCV mono-infection, there are also infection-specific differences regarding perturbations of the NK cell pool. As an example, expression of the inhibitory C-type lectin receptor NKG2A has repeatedly been demonstrated to be increased in chronic hepatitis C[24,32], whereas HIV infection is associated with a decreased frequency of NKG2A[10,33]. Furthermore, Meier et al were able to detect significant differences in the production of IFN-γ between HIV and HCV[16]. Moreover, anti-viral[11,34,35] as well as anti-fibrotic[15] NK cell functions have been shown to be impaired in HIV patients even in the context of effective cART.

Although a large number of studies analyzed the impact of HIV and HCV mono-infection, respectively, on phenotype and functions of NK cells, little is known regarding alterations of this lymphocyte subset in HIV patients chronically co-infected with HCV.

Here, we show that HIV/HCV co-infection is associated with a significant dysregulation of the circulating NK cell pool, and present data suggesting that dysregulation mainly reflects alterations observed in HIV mono-infection.

## Materials and methods

### Patients

Our cross-sectional study was conducted among patients who attended the outpatient clinic of the Department of Internal Medicine I at the university hospital in Bonn, Germany, between the beginning of 2013 and the end of 2015. Gender, age, CD4^+^ T-cell counts, HIV-1 and HCV RNA viral load as well as liver enzyme levels were retrieved from patients’ files. A total of 108 patients, including 35 HIV mono-infected, 39 HCV mono-infected and 34 HIV/HCV co-infected individuals, all from the Cologne/Bonn area in Germany, were enrolled into this study. All HIV(+) patients were under effective cART, containing a combination of two nucleoside/nucleotide reverse transcriptase inhibitors (NRTI) and either a ritonavir-boosted protease inhibitor (PI) or a non-NRTI (NNRTI), with HIV RNA loads persistently below the level of detection. As a control group 43 healthy HIV(-)/HCV(-) individuals were studied. A detailed description of patient characteristics is given in Table 1 (Table 1). Informed written consent was obtained from all patients. The study had been approved by the local ethics committee of the University of Bonn (Bonn, Germany).

**Table 1:** Patient characteristics.

					Group comparison (*P* value)		
	Healthy	HIV	HCV	HIV/HCV	HIV vs. HIV/HCV	HCV vs. HIV/HCV	HIV vs. HCV
**Number**	43	35	39	34			
**Male sex**^**a)**^	22 (51.2%)	22 (62.9%)	27 (69.2%)	30 (88.2%)	0.01	n.s.	n.s.
**Age (years)**^**b)**^	33 (21–55)	44 (27–68)	48 (21–78)	45 (30–58)	n.s.	n.s.	n.s.
**Clinical data**							
AST (U/L)^b)^	n/a	29 (15–73)	79 (18–247)	64 (19–362)	0.003	n.s.	<0.0001
ALT (U/L) ^b)^	n/a	33 (10–96)	127 (11–705)	138 (24–984)	0.0001	n.s.	<0.0001
gammaGT (U/L)^b)^	n/a	81 (24–347)	116 (13–600)	113 (29–413)	n.s.	n.s.	n.s.
Bilirubin (mg/dL)^b)^	n/a	0.7 (0.25–1.7)	0.7 (0.4–1.2)	0.7 (0.26–2.76)	n.s.	n.s.	n.s.
MELD score^b)^	n/a	7 (6–9)	6 (6–6)	6 (6–7)	n.s.	n.s.	n.s.
**HIV status**							
HIV RNA non-detectable (cART)^a)^^c)^	n/a	35 (100%)	n/a	34 (100%)			
CD4 (cells/μL)^b)^	n/a	643 (181–1199)	n/a	615 (34–1296)	n.s.	-	-
CD8 (cells/μL)^b)^	n/a	1166 (366–2163)	n/a	1008 (254–2249)	n.s.	-	-
**HCV status**							
HCV RNA (x10^6^ IU/mL)^b)^	n/a	n/a	3.1 (<0.01–21.5)	4.5 (0.13–26.1)	-	n.s.	-
**HCV genotype**							
Genotype 1^a)^	n/a	n/a	30 (76.9%)	21 (61.8%)	-	n.s.	-
Genotype 2^a)^	n/a	n/a	0	0	-	n.s.	-
Genotype 3^a)^	n/a	n/a	5 (12.8%)	2 (5.9%)	-	n.s.	-
Genotype 4^a)^	n/a	n/a	1 (2.6%)	4 (11.8%)	-	n.s.	-
more than one^a)^	n/a	n/a	1 (2.6%)	2 (5.9%)	-	n.s.	-
unknown^a)^	n/a	n/a	2 (5.1%)	5 (14.7%)	-	n.s.	-

^a)^ number of cases (% of total)

^b)^ mean (range)

^c)^ cART contains a combination of two nucleoside/nucleotide reverse transcriptase inhibitors (NRTI) and either a ritonavir-boosted protease inhibitor (PI) or a non-NRTI (NNRTI)

“MELD” = Model for End-Stage Liver Disease; “n/a” = not available / not analyzed; “n.s.” = non-significant

### Isolation of PBMC

Peripheral blood mononuclear cells (PBMC) were isolated from venous blood by Ficoll-Paque (PAA, Germany) density gradient centrifugation as described elsewhere[36] and cryopreserved. PBMC were cultured in RPMI medium (PAA, Germany) supplemented with 10% fetal calf serum (FCS) (Biochrom, Germany) and 1% penicillin/streptomycin (PAA, Germany) at 37°C and 5% CO_2_.

### HuH7 HCV_replicon_ cells

HuH7 HCV_replicon_ cells were kindly provided by V. Lohmann and R. Bartenschlager (University of Heidelberg, Heidelberg, Germany). Cells were grown in high-glucose Dulbecco’s modified Eagle’s medium (DMEM) (4.5 g/L) supplemented with glutamine (PAA Laboratories GmbH, Cölbe, Germany), 10% fetal calf serum (FCS), 1% nonessential amino acids (Biochrom AG, Berlin, Germany) and 1% penicillin/streptomycin (PAA, Germany). Blasticidin S hydrochloride (3 μg/mL) and G418 (1 mg/mL; PAA, Germany) were added to cells containing subgenomic replicons. HuH7 HCV_replicon_ cells were passaged twice a week and were seeded at a dilution of 1:3.

### I) NK cell analysis: Frequency and phenotype

**Fig 1:**
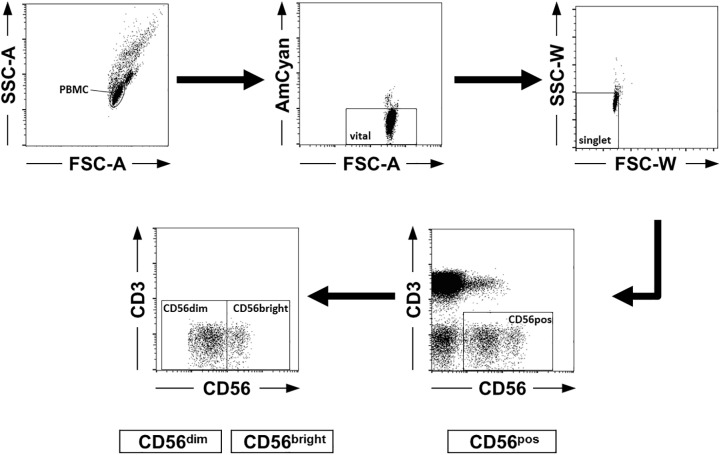
Gating strategy for identification of natural killer (NK) cells. Representative gating strategy for the identification of CD56^dim^ and CD56^bright^ NK cell subsets. 1^st^ upper panel: The population of peripheral blood mononuclear cells (PBMC) was localized by forward (FSC) and side scatter (FSC) gating using areal scaling (FSC-A, SSC-A). 2^nd^ upper panel: Dead cells were excluded by use of Zombie Aqua Fixable Viability Kit (Biolegend, Fell, Germany). 3^rd^ upper panel: Cell aggregates were eliminated by width parameters for FSC and SSC (FSC-W, SSC-W). Lower panels: NK cells were defined as CD3^-^ CD56^+^ cell population. CD56^dim^ and CD56^bright^ NK cells were discriminated by low versus high expression of CD56.

Cryopreserved PBMC were thawed, washed with phosphate buffered saline (PBS) and stained with anti-CD3 and anti-CD56 fluorochrome-labeled antibodies for detection of NK cells by fluorescence activated cell sorting (FACS). In addition, cells were stained with different combinations of the following fluorochrome-labeled antibodies: anti-CD27, anti-CD57, anti-CD62L, anti-CD69, anti-CD127, anti-NKG2A, anti-NKG2C, anti-NKG2D, anti-NKp30, anti-NKp46, anti-CD107a and anti-INF-γ (Table 2). Isotype controls were used to set up gates and to determine positive and negative populations, respectively (S1 Fig). Dead cells were excluded by use of Zombie Aqua Fixable Viability Kit (Biolegend, Fell, Germany). After incubation of the cells with 1 to 10 μl of antibody solution (depending on antibody) and washing with PBS, samples were analyzed on a FACSCanto II flow cytometer using CellQuest Pro (BD Biosciences) and FlowJo 7.5 software packages (TreeStar Inc., Ashland, OR, USA). To ensure consistent cytometer settings Rainbow Calibration Particles (Biolegend, Fell, Germany) were used. Fig 1 depicts the complete gating strategy for identification of NK cells as well as CD56^dim^ and CD56^bright^ subsets (Fig 1).

**Table 2:** Monoclonal antibodies used for flow cytometry.

Antigen	Fluorochrome	Clone	Isotype	Company	Catalog no.
CD3	PerCP	SK7	mouse IgG1, κ	Biolegend, Fell, Germany	344814
CD3	APC-Cy7	HIT3a	mouse IgG2a, κ	Biolegend, Fell, Germany	300318
CD56	APC	HCD56	mouse IgG1, κ	Biolegend, Fell, Germany	318310
CD56	FITC	NCAM16.2	mouse IgG2b, κ	BD Biosciences	345811
CD27	PE	O323	mouse IgG1, κ	Biolegend, Fell, Germany	302808
CD62L	FITC	DREG-56	mouse IgG1, κ	Biolegend, Fell, Germany	304804
CD127	FITC	HIL-7R-M21	mouse IgG1, κ	BD Biosciences	560549
CD57	FITC	HCD57	mouse IgM, κ	Biolegend, Fell, Germany	322306
NKG2A	PE	131411	mouse IgG2a	R&D Systems, USA	FAB1059P
NKG2C	PerCP	134591	mouse IgG1	R&D Systems, USA	FAB138C
NKG2D	PE	1D11	mouse IgG1, κ	Biolegend, Fell, Germany	320806
NKp30	PE	P30-15	mouse IgG1, κ	Biolegend, Fell, Germany	325208
NKp46	APC	9E2	mouse IgG1, κ	Biolegend, Fell, Germany	331918
CD69	PE-Cy7	FN50	mouse IgG1, κ	Biolegend, Fell, Germany	310912
CD107a	FITC	H4A3	mouse IgG1, κ	BD Biosciences	555800
IFN-γ	PE	25723	mouse IgG2B	R&D Systems, USA	IC285P

### II) NK cell analysis: CD107a degranulation assay

NK cell degranulation was assessed in response to HuH7 HCV_replicon_ cells. To this end, cryopreserved PBMC were thawed and cultured at 37°C in complete medium without any exogenously added cytokines. After 12 hours, PBMC were washed, resuspended and pre-stimulated with recombinant human interleukin (rhIL)-2 (25 U/mL; R&D Systems, USA). Then, cells were co-incubated with HuH7 HCV_replicon_ cells at an effector/target (E:T) ratio of 1:1 in the presence of anti-CD107a to assess degranulation, as described before[37].

### III) NK cell analysis: IFN-γ secretion

Cryopreserved PBMC were thawed, pre-stimulated with rhIL-2 (25 U/mL; R&D Systems, USA) followed by co-incubation with HuH7 HCV_replicon_ cells at an E:T ratio of 1:1 at 37°C for 5 hours. Brefeldin A (10 μg/mL; Sigma-Aldrich, St. Louis, MO, USA) was added after 1 hour of co-culture. Next, cells were harvested and washed, followed by intracellular staining with anti-IFN-γ and flow cytometry analysis.

### Statistical analysis

Frequencies of cell sub-populations between the different cohorts were compared using Kruskal-Wallis tests. Test multiplicity was controlled by a false discovery rate (FDR) procedure accounting for dependency among statistical tests [38]. FDR-adjusted *P* values <0.05 were considered statistically significant. Statistical analyses were performed using GraphPad Prism Version 5.0a (GraphPad Software Inc, San Diego, CA, USA) and the SPSS 17.0 statistical package (SPSS, Chicago, IL, USA).

## Results

### Frequency of NK cells in HIV/HCV co-infection

**Fig 2:**
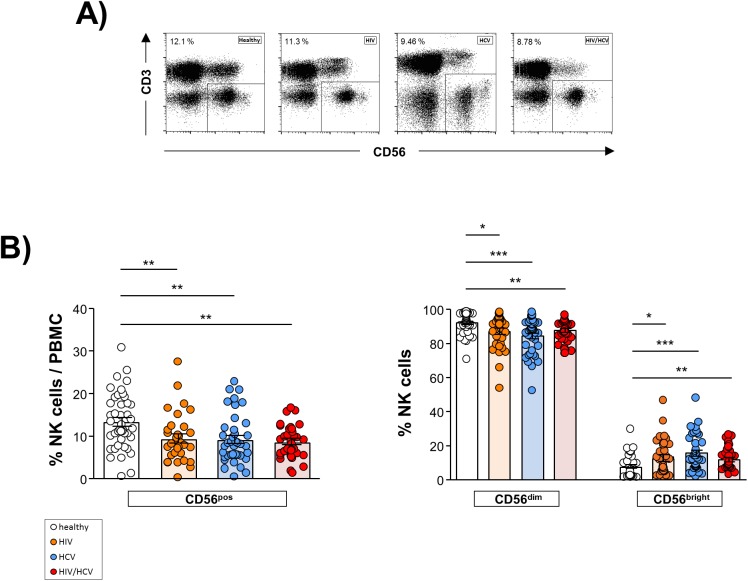
Frequency of circulating natural killer (NK) cells. **A**) Representative dot plots of flow cytometric analyses. **B**) 1^st^ panel: Frequency of circulating NK cells, displayed as percentage of vital, singlet peripheral blood mononuclear cells (PBMC) in healthy donors (n = 43), HIV mono-infected patients (HIV; n = 35), HCV mono-infected individuals (HCV; n = 39) and co-infected patients (HIV/HCV; n = 34). 2^nd^ panel: Distribution of CD56^dim^ and CD56^bright^ NK cells, displayed as percentage of total NK cells in healthy donors (n = 43), HIV (n = 35), HCV (n = 39) and HIV/HCV (n = 34). * p≤0.05, ** p≤0.01, *** p≤0.001, “n.s.” non-significant.

First, we analyzed the frequency of NK cells among our study cohorts (Fig 2A). The frequency of circulating NK cells, assessed as percentage of CD3^-^ CD56^+^ cells among PBMC, was significantly lower in HIV/HCV co-infection compared to healthy controls but did not differ significantly from that observed in HIV and HCV mono-infected patients, respectively (Fig 2B, left panel). HIV/HCV co-infection was associated with an increased frequency of CD56^bright^ NK cells, whereas its proportion of CD56^dim^ and CD56^bright^ cells was similar to both mono-infected groups, respectively (Fig 2B, right panel).

### Expression of maturation/differentiation markers in HIV/HCV co-infection

**Fig 3:**
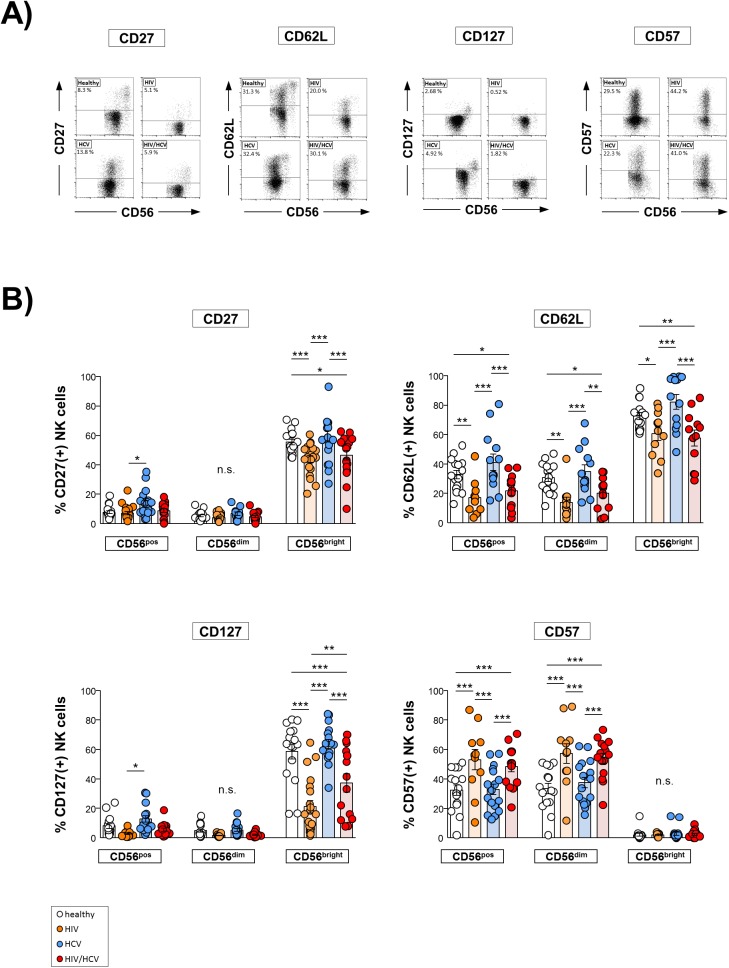
Surface expression of maturation/differentiation markers. **A**) Representative dot plots of flow cytometric analyses. **B**) Expression of maturation/differentiation markers was determined by flow cytometry on total (CD56^pos^), CD56^dim^ and CD56^bright^ natural killer (NK) cells. 1^st^ upper panel: Expression of CD27 in healthy donors (n = 18), HIV mono-infected patients (HIV; n = 20), HCV mono-infected individuals (HCV; n = 17) and HIV/HCV co-infected patients (HIV/HCV; n = 17). 2^nd^ upper panel: Expression of CD62L in healthy donors (n = 15), HIV (n = 11), HCV (n = 13) and HIV/HCV (n = 12). 1^st^ lower panel: Expression of CD127 in healthy donors (n = 16), HIV (n = 17), HCV (n = 16) and HIV/HCV (n = 14). 2^nd^ lower panel: expression of CD57 in healthy donors (n = 16), HIV (n = 11), HCV (n = 17) and HIV/HCV (n = 15). * p≤0.05, ** p≤0.01, *** p≤0.001, “n.s.” non-significant.

Next, we studied the NK cell maturation/differentiation status in our cohorts (Fig 3A). Compared to healthy controls we found HIV/HCV co-infection to be associated with a decreased frequency of CD56^bright^ NK cells expressing markers characteristic for less mature NK cells, such as CD27, CD62L and CD127 (Fig 3B), whereas the proportion of CD57^+^ NK cells was significantly increased (Fig 3B, lower right panel). Similar observations were made in HIV mono-infected patients. In contrast, NK cells from HCV mono-infected individuals displayed an expression of CD27, CD62L, CD127 and CD57 molecules similar to that observed in healthy controls.

### Expression of NK cell receptors in HIV/HCV co-infection

**Fig 4:**
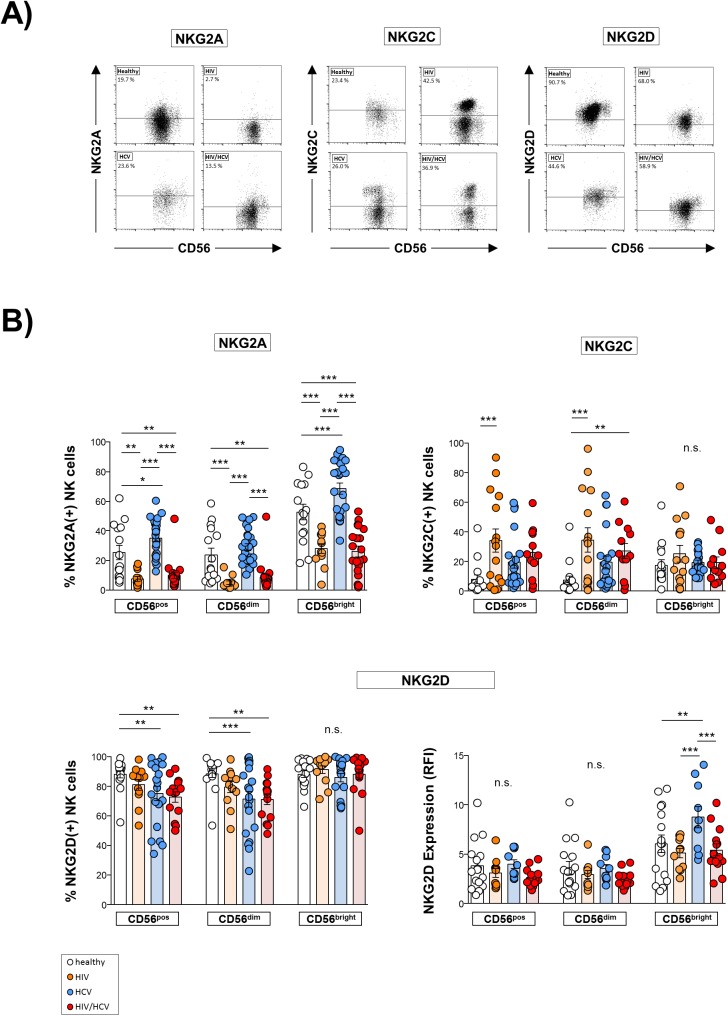
Surface expression of natural killer (NK) cell receptors (NKR). **A**) Representative dot plots of flow cytometric analyses. **B**) Expression of NKR was determined by flow cytometry on total (CD56^pos^), CD56^dim^ and CD56^bright^ NK cells. 1^st^ upper panel: Expression of the C-type lectin receptor NKG2A in healthy donors (n = 15), HIV mono-infected patients (HIV; n = 12), HCV mono-infected individuals (HCV; n = 25) and HIV/HCV co-infected patients (HIV/HCV; n = 20). 2^nd^ upper panel: Expression of NKG2C in healthy controls (n = 15), HIV (n = 15), HCV (n = 21) and HIV/HCV (n = 12). 1^st^ lower panel: Expression of NKG2D in healthy persons (n = 16), HIV (n = 12), HCV (n = 20) and HIV/HCV (n = 14). 2^nd^ lower panel: Surface expression density (RFI) of NKG2D in healthy persons (n = 16), HIV (n = 11), HCV (n = 10) and HIV/HCV (n = 14). * p≤0.05, ** p≤0.01, *** p≤0.001, “n.s.” non-significant.

**Fig 5:**
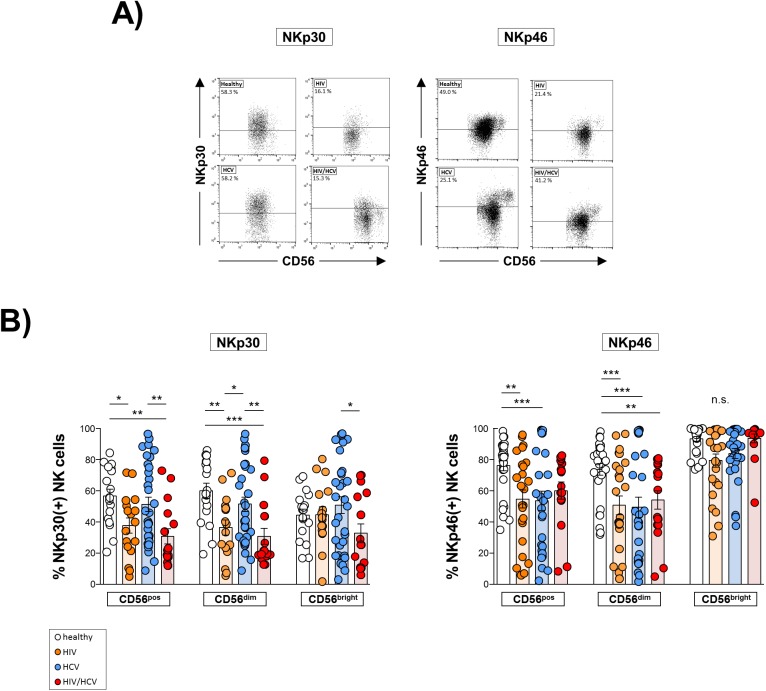
Surface expression of natural cytotoxicity receptors (NCR). **A**) Representative dot plots of flow cytometric analyses. **B**) Expression of NCR was determined by flow cytometry on total (CD56^pos^), CD56^dim^ and CD56^bright^ NK cells. 1^st^ panel: Expression of NKp30 in healthy donors (n = 18), HIV mono-infected patients (HIV; n = 17), HCV mono-infected individuals (HCV; n = 32) and HIV/HCV co-infected patients (HIV/HCV; n = 16). 2^nd^ panel: Expression of NKp46 in healthy controls (n = 28), HIV (n = 25), HCV (n = 29) and HIV/HCV (n = 17). * p≤0.05, ** p≤0.01, *** p≤0.001, “n.s.” non-significant.

Then, we analyzed the expression of NK cell receptors (NKR) which have been shown to critically regulate NK cell functions (Figs 4A and 5A). As is shown in Fig 4B we found HIV mono- as well as HIV/HCV co-infection to be associated with a significantly lower frequency of NKG2A-expressing NK cells than healthy controls whereas patients with chronic hepatitis C displayed a significantly higher frequency of NKG2A^+^ NK cells compared to all other studied groups (Fig 4B, upper left panel). Moreover, we found the frequency of CD56^dim^ NKG2C^+^ NK cells to be significantly higher in HIV/HCV co- and HIV mono-infected patients than in healthy individuals (Fig 4B, upper right panel). In addition, we observed the frequency of NKG2D^+^ NK cells to be reduced in co-infected patients (Fig 4B, lower left panel), whereas NKG2D surface expression density (RFI) was not affected (Fig 4B, lower right panel). Similar observations were made in patients mono-infected with HCV. However, CD56^bright^ NK cells in HCV patients displayed an increased surface expression density of NKG2D. With respect to expression of the natural cytotoxicity receptors (NCR) NKp30 and NKp46 we found HIV(+) patients to display a significantly lower frequency of NKp30^+^ NK cells than HCV mono-infected patients and healthy controls, irrespective of HCV co-infection (Fig 5B, left panel), whereas the frequency of NKp46^+^ NK cells was reduced in all patient cohorts (Fig 5B, right panel).

### HIV and HCV infection affect NK cell functionality

**Fig 6:**
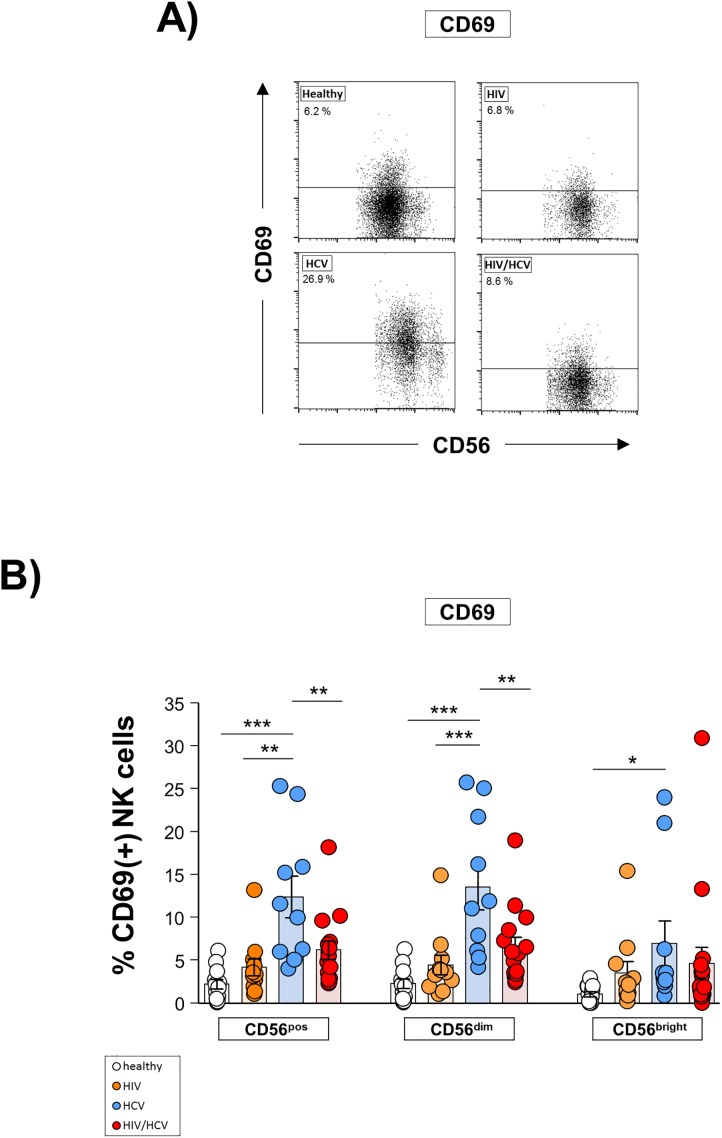
HIV and HCV infection affect natural killer (NK) cell activation status. **A**) Representative dot plots of flow cytometric analyses. **B**) Expression of the activation marker CD69 was determined by flow cytometry on total (CD56^pos^), CD56^dim^ and CD56^bright^ NK cells in healthy donors (n = 12), HIV mono-infected patients (HIV; n = 11), HCV mono-infected individuals (HCV; n = 10) and HIV/HCV co-infected patients (HIV/HCV; n = 15). * p≤0.05, ** p≤0.01, *** p≤0.001, “n.s.” non-significant.

**Fig 7:**
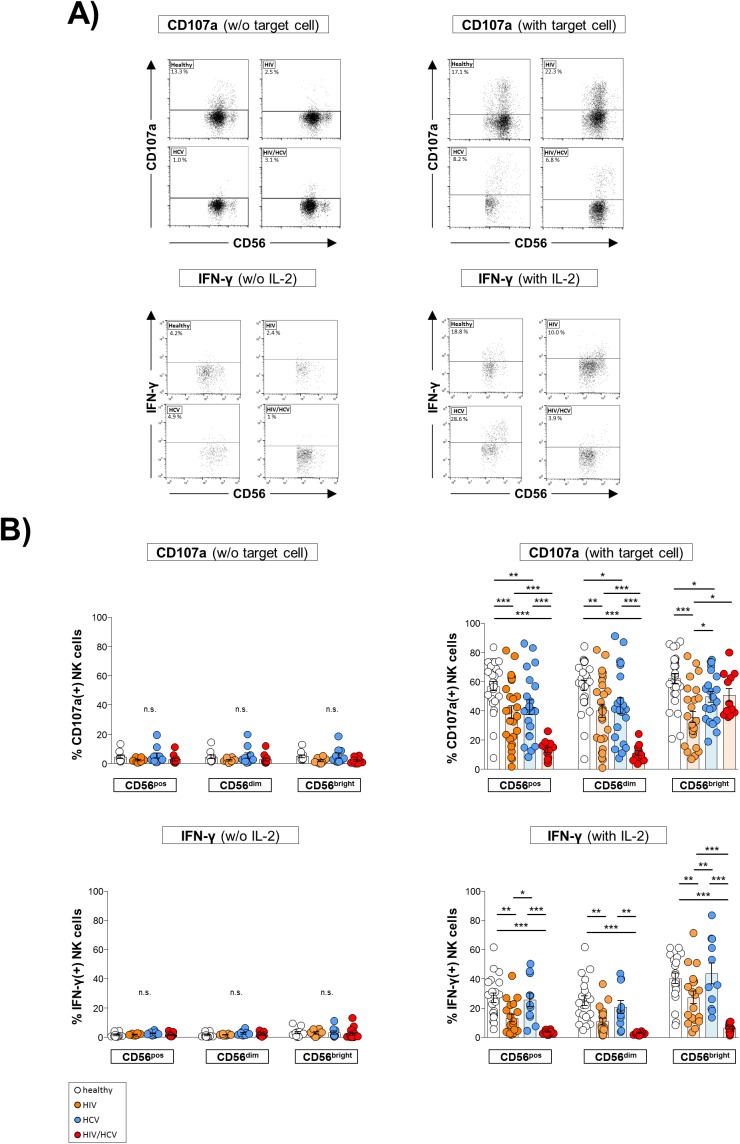
HIV and HCV infection affect natural killer (NK) cell functionality. **A**) Representative dot plots of flow cytometric analyses. **B**) Expression of markers was determined by flow cytometry on total (CD56^pos^), CD56^dim^ and CD56^bright^ NK cells. 1^st^ upper panel: Surface expression of CD107a in healthy donors (n = 10), HIV mono-infected patients (HIV; n = 8), HCV mono-infected individuals (HCV; n = 10) and HIV/HCV co-infected patients (HIV/HCV; n = 12). 2^nd^ upper panel: Surface expression of CD107a following co-incubation of peripheral blood mononuclear cells (PBMC) with HuH7 HCV_replicon_ cells in healthy controls (n = 27), HIV (n = 27), HCV (n = 21) and HIV/HCV (n = 12). 1^st^ lower panel: Unstimulated PBMC were co-cultured with HuH7 HCV_replicon_ cells. Production of IFN-γ was determined by intracellular staining of NK cells from healthy controls (n = 12), HIV (n = 8), HCV (n = 10) and HIV/HCV (n = 19). 2^nd^ lower panel: rhIL-2 stimulated PBMC were co-cultured with HuH7 HCV_replicon_ cells. Production of IFN-γ was determined in healthy controls (n = 20), HIV (n = 19), HCV (n = 11) and HIV/HCV (n = 12). * p≤0.05, ** p≤0.01, *** p≤0.001, “n.s.” non-significant.

Finally, we studied the NK cell activation status (Fig 6A) and functional capacity (Fig 7A). As is depicted in Fig 6B, the frequency of NK cells expressing the activation marker CD69 was not different in HIV/HCV co-infected patients from healthy controls, while HCV mono-infected patients showed the highest frequency in comparison to all other groups (Fig 6B). With respect to NK cell function, we observed degranulation of rhIL-2-stimulated NK cells following co-incubation with HuH7 HCV_replicon_ cells to be significantly impaired in all virus-infected patient groups as compared to healthy controls (Fig 7B, upper right panel). Of note, this defect was most pronounced in HIV/HCV co-infected patients, which were found to have a significantly lower frequency of CD107a^+^ NK cells than patients mono-infected with HIV and HCV, respectively. No such differences were seen when NK cell degranulation was studied in the absence of target cells (Fig 7B, upper left panel). Analyzing IFN-γ production of rhIL-2 stimulated NK cells we observed a significantly reduced percentage of IFN-γ^+^ NK cells in HIV mono-infected patients compared to healthy and HCV mono-infected individuals (Fig 7B, lower right panel). However, this functional defect was strongest in HIV infected patients co-infected with HCV. IFN-γ production of unstimulated NK cells was similar between the four study groups (Fig 7B, lower left panel).

## Discussion

NK cells have been shown to importantly contribute to anti-HCV immune responses and to modulate HCV-associated liver fibrosis[10,12,15,29]. However, little is known regarding the effects of HIV/HCV co-infection on functions and phenotype of NK cells. Here, we show that HIV/HCV co-infection is associated with a significant dysregulation of the NK cell pool, including both phenotypic as well as functional alterations.

NK cell development is considered to proceed sequentially from CD56^bright^ to CD56^dim^ NK cells[39–41]. This process is associated with a gradual decrease in CD27, CD62L and CD127, all highly expressed on circulating CD56^bright^ cells, together with a progressive increase in receptors characteristic of mature cells, such as CD57[18,42–50]. Of note, we found HIV/HCV co-infection to be associated with a shift towards a more mature NK cell phenotype as frequency of NK cells expressing CD27, CD62L and CD127 was significantly lower than in healthy controls, whereas the proportion of mature CD57 expressing NK cells was increased. Moreover, we found the frequency of NK cells positive for NKG2A, which has been shown to be highly expressed on less mature NK cells but to display a stepwise decrease during terminal NK cell differentiation[42,43,48], to be significantly lower in co-infected patients than in healthy individuals. Similar observations were made in HIV mono-infected patients. This is in line with findings by Hong et al. who demonstrated a decline of less differentiated NK cells in chronic HIV mono-infection[44]. The exact mechanisms underlying HIV-driven rapid NK cell maturation are only incompletely understood but may at least in part be the result of HIV-induced ongoing activation of T- and B-cells[51–57]. Of note, markers of immune activation have been shown to be associated with plasma levels of microbial products in HIV infection. Thus, increased levels of circulating microbial products, resulting from microbial translocation, have been proposed to represent a major cause of HIV-induced chronic T- and B-cell activation[51]. Thus, similar mechanisms may also play a role with respect to NK cells. NK cell maturation has been also observed in other viral diseases such as infection with the cytomegalovirus (CMV) and, thus, is not specific to HIV(+) individuals[47,58]. In HCV mono-infected patients, however, such a mechanism seems to be of minor if any relevance because the NK cell maturation status did not differ significantly from that found in healthy controls.

Several reports demonstrated significant alterations of the NK cell receptor expression in HIV and HCV mon-infection, respectively[11,21,24–26,28,30,59–62]. Of note, some of these perturbations have been found to differ significantly between both viral infections. The most prominent example relates to the expression of the inhibitory NKG2A receptor, which has been shown to be up-regulated in chronic HCV mono-infection[24,25,28,30,63] but reduced in HIV mono-infection[11,62,63]. These findings could be confirmed in the present study. More importantly, we found HIV/HCV co-infection to be associated with low expression of NKG2A similar to that seen in patients with HIV mono-infection. In addition, we found the expression of the activating receptors NKp30 and NKp46 to be down-regulated in both HIV mono-infected as well as in HIV/HCV co-infected patients. This is in line with earlier reports which also demonstrated a reduced surface expression of activating NK cell receptors in HIV(+) patients[59,60]. Activation of NK cells has been proposed to result in down-regulation of NKp30 and NKp46[21,26]. Thus, low expression of NKp30 and NKp46 in HIV mono-infected and HIV/HCV co-infected patients might be due to HIV-induced NK cell activation. However, NK cell expression of the activation marker CD69 was not altered in HIV infected patients, irrespective of HCV co-infection, while elevated in HCV mono-infected patients, which rather argues against such a mechanism, since NKp30 expression in HCV mono-infected patients did not differ from that seen in healthy controls but was significantly higher than in HIV(+) individuals.

With respect to NK cell functions we observed HIV mono-infection to be associated with significantly impaired IFN-γ production, which is in line with previous reports[34,64,65]. No such dysregulation of NK cell activity could be found in HCV mono-infection, whereas HIV/HCV co-infected individuals displayed the lowest IFN-γ production. The degranulation rate of NK cells was reduced in all infected individuals while, similar to IFN-γ production, the most pronounced reduction could be found in co-infection. The exact mechanisms involved in impaired NK cell functions in HIV/HCV co-infection remain to be clarified. In HIV mono-infected patients, however, perturbed NK cell maturation as well as altered NK cell receptor expression have been suggested to cause dysfunctionality of NK cells[59,60,66]. Thus, it is conceivable that similar mechanisms might also play a role in co-infected patients.

Our study has several limitations. First, we only included HIV(+) patients under effective therapy and, thus, the effect of ongoing HIV replication on NK cells could not be assessed. Second, the number of cells that were available for our study was limited. Therefore, we could not analyze all markers in all patients. Moreover, a number of surface molecules important for NK cell functions and/ or differentiation, such as killer cell immunglobulin-like receptors (KIR), could not be tested. Third, we focused on CD56^+^ cells but did not examine the CD56^-^ counterpart. Accordingly, further studies are needed to characterize the full extent of alterations in the NK cell compartment in HIV/HCV co-infection.

Taken together, our data indicate that HIV/HCV co-infection is associated with significant alterations of the NK cell pool, which mainly reflect alterations observed in HIV mono-infection.

## Supporting information

S1 Fig Expression of measured markers. Peripheral blood mononuclear cells (PBMC) were stained with different fluorochrome-labeled or isotype-matched antibodies and analyzed on a flow cytometer. Blue histogram profile indicates the isotype control, and red histogram indicates the specific antibody.
(TIF)

S1 Table Original data shown in Fig 2B (XLSX)

S2 Table Original data shown in Fig 3B (XLSX)

S3 Table Original data shown in Fig 4B (XLSX)

S4 Table Original data shown in Fig 5B (XLSX)

S5 Table Original data shown in Fig 6B (XLSX)

S6 Table Original data shown in Fig 7B (XLSX)
