# Supplementary figures and images for: Alterations of the NK cell pool in HIV/HCV co-infection
Source: PLoS One. 2017 Apr 5;12(4):e0174465. doi: 10.1371/journal.pone.0174465 (PMC5381812; doi:10.1371/journal.pone.0174465)

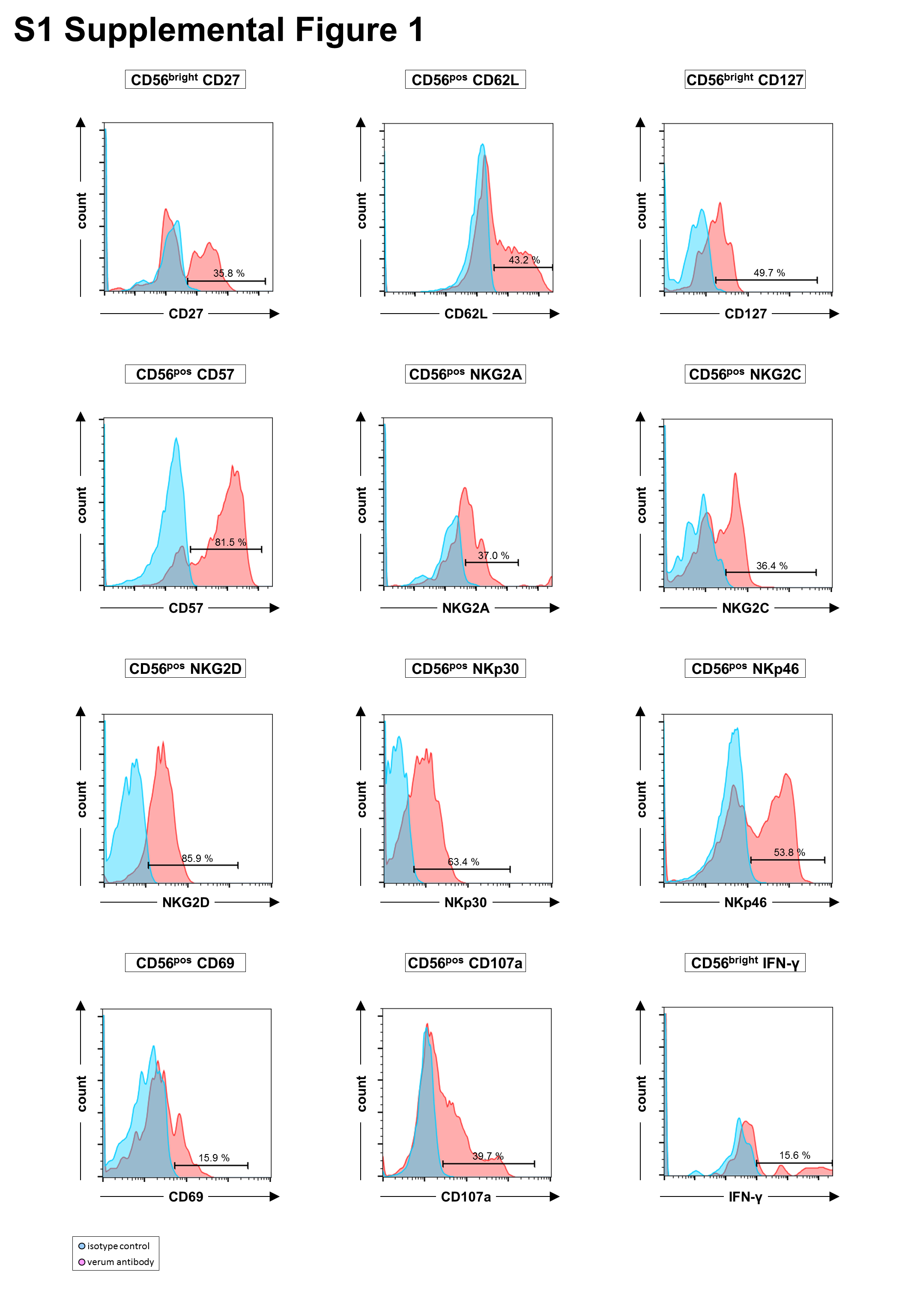

Supplement: S1 Fig — Peripheral blood mononuclear cells (PBMC) were stained with different fluorochrome-labeled or isotype-matched antibodies and analyzed on a flow cytometer. Blue histogram profile indicates the isotype control, and red histogram indicates the specific antibody. (TIF) [file pone.0174465.s001.tif]
